# Supplementary material for: Data driven approach to characterize rapid decline in autosomal dominant polycystic kidney disease
Source: PLoS One. 2024 Jun 5;19(6):e0298484. doi: 10.1371/journal.pone.0298484 (PMC11152279; doi:10.1371/journal.pone.0298484)
Supplement: S1 File — (DOCX) [file pone.0298484.s002.docx]

**Supplementary Tables:**

**Supplemental Table 1. Mean Lab Values Among Patients in the Non-Rapid Decliner vs. Rapid Decliner Group**

| Laboratory* | Overall | Non-Rapid Decliner | Rapid Decliner | SMD | Missing (%) |
| --- | --- | --- | --- | --- | --- |
|  | (N=1744) | (N=1619) | (N=125) |  |  |
| Creatinine (mg/dL) | 1.3 (0.6) | 1.3 (0.6) | 1.2 (0.6) | 0.15 | 0 |
| Sodium (mmol/L) | 138.9 (2.9) | 139.0 (2.8) | 138.1 (3.3) | 0.297 | 8.3 |
| Potassium (mg/dL) | 4.1 (0.5) | 4.1 (0.5) | 4.0 (0.4) | 0.212 | 7.2 |
| Chloride (mEq/L) | 103.9 (3.4) | 103.9 (3.4) | 103.8 (3.5) | 0.023 | 9.0 |
| Bicarbonate (mEq/L) | 26.7 (2.9) | 26.8 (2.9) | 25.9 (2.7) | 0.331 | 9.1 |
| Blood Urea Nitrogen (mg/dL) | 20.5 (12.0) | 20.7 (12.2) | 18.4 (9.1) | 0.216 | 19.7 |
| Phosphorus (mg/dL) | 3.5 (0.8) | 3.5 (0.7) | 3.5 (1.0) | 0.049 | 59.7 |
| Calcium (mg/dL) | 9.3 (0.6) | 9.3 (0.6) | 9.3 (0.7) | 0.09 | 46.6 |
| PTH (pg/mL) | 77.4 (59.1) | 78.0 (60.5) | 68.3 (35.2) | 0.196 | 76.4 |
| Vitamin D (ng/mL) | 30.5 (12.9) | 30.7 (12.8) | 27.3 (13.2) | 0.262 | 76.0 |
| Hemoglobin (g/dL) | 13.5 (1.7) | 13.6 (1.7) | 13.1 (1.6) | 0.268 | 7.1 |
| Platelets (1000/µL) | 242.9 (74.1) | 243.1 (74.2) | 240.1 (73.1) | 0.041 | 9.1 |
| WBC (1000/µL) | 15.5 (156.8) | 16.1 (163.0) | 8.2 (3.1) | 0.068 | 9.2 |
| Iron (mcg/dL) | 76.1 (36.9) | 76.9 (37.2) | 65.7 (31.7) | 0.323 | 72.7 |
| Transferrin Saturation (mcg/dL) | 24.4 (11.6) | 24.6 (11.8) | 20.9 (8.5) | 0.36 | 73.3 |
| Ferritin (ng/dL) | 187.4 (211.8) | 190.1 (215.8) | 155.6 (153.4) | 0.184 | 76.5 |
| Albumin (g/dL) | 3.8 (0.6) | 3.8 (0.6) | 3.6 (0.6) | 0.329 | 58.8 |
| Uric Acid (mg/dL) | 6.8 (2.1) | 6.8 (2.1) | 7.0 (2.2) | 0.101 | 75.5 |
| ALT (U/L) | 26.3 (26.8) | 26.5 (27.5) | 24.2 (13.6) | 0.108 | 22.3 |
| AST (U/L) | 28.2 (25.4) | 28.4 (26.2) | 26.0 (12.7) | 0.117 | 63.5 |
| ALP (IU/L) | 78.2 (52.1) | 77.7 (51.4) | 85.6 (60.7) | 0.14 | 58.7 |
| Total Bilirubin (mg/dL) | 0.9 (1.4) | 0.9 (1.5) | 0.8 (0.7) | 0.039 | 59.6 |
| ESR (mm/h) | 27.6 (24.9) | 27.6 (24.7) | 26.5 (28.6) | 0.041 | 88.0 |
| CRP (mg/L) | 27.1 (54.0) | 28.6 (55.9) | 10.6 (20.6) | 0.427 | 96.4 |
| Glucose (mg/dL) | 105.5 (32.9) | 104.6 (30.4) | 116.0 (55.1) | 0.257 | 21.0 |
| Hemoglobin A1C | 6.0 (1.0) | 6.0 (1.0) | 6.1 (0.9) | 0.117 | 59.1 |
| Total cholesterol (mg/dL) | 184.7 (45.1) | 184.5 (44.4) | 187.7 (55.2) | 0.064 | 24.4 |
| HDL (mg/dL) | 48.5 (15.3) | 48.8 (15.0) | 44.2 (18.3) | 0.274 | 24.9 |
| LDL (mg/dL) | 108.0 (34.3) | 108.0 (34.1) | 107.1 (36.2) | 0.027 | 26.4 |
| Triglyceride (mg/dL) | 152.0 (156.6) | 148.2 (146.2) | 208.8 (263.1) | 0.285 | 38.6 |
| LDH (units/L) | 193.9 (145.4) | 198.5 (154.1) | 163.6 (56.5) | 0.301 | 94.7 |
| Urine Protein | 490.1 (835.1) | 459.8 (856.4) | 719.2 (626.9) | 0.346 | 92.1 |
| Urine Microalbumin/Creatinine Ratio | 278.3 (740.3) | 248.5 (677.0) | 719.6 (1305.9) | 0.453 | 61.1 |
| Urine Protein/Creatinine Ratio | 0.50 (1.0) | 0.4 (0.9) | 0.9 (1.5) | 0.351 | 81.7 |
| Urine Phosphorus | 22.1 (124.7) | 23.4 (128.3) | 1.0 (0.4) | 0.247 | 97.9 |
| Urine Calcium | 119.4 (91.7) | 125.6 (91.6) | 45.5 (59.9) | 1.036 | 97.0 |
| Positive Urine Protein | 0.4 (0.5) | 0.4 (0.5) | 0.6 (0.5) | 0.481 | 13.8 |
| Positive Urine WBC | 0.4 (0.5) | 0.4 (0.5) | 0.4 (0.5) | 0.033 | 21.7 |
| Positive Urine RBC | 0.4 (0.5) | 0.4 (0.5) | 0.4 (0.5) | 0.038 | 34.9 |

*Data shown are means (standard deviation)

|  | Total f/u time (years) | Median f/u time (years) median (IQR) | # of events | IR/1000 person-years (95% CI*) | Time to event (years) median (IQR) | Hazard ratio (95% CI) |
| --- | --- | --- | --- | --- | --- | --- |
| **ESKD^1^, N=1744** |  |  |  |  |  |  |
| Rapid decliner (n=125) | 643 | 4.2 (2.9, 6.8) | 59 | 91.7 (75.2, 111.8) | 4.8 (3.3, 7.4) | 6.3 (4.7, 8.4) |
| Non-rapid decliner (n=1619) | 9961 | 5.2 (2.3, 9.1) | 169 | 17.0 (14.7, 19.6) | 6.2 (3.4, 10.1) | Reference |
| **ESKD before age 53^2^, N=927** |  |  |  |  |  |  |
| Rapid decliner (n=94) | 567 | 4.8 (3.2, 8.1) | 40 | 70.5 (54.0, 92.0) | 4.8 (3.2, 8.1) | 10.5 (6.8, 16.3) |
| Non-rapid decliner (n=833) | 5470 | 5.4 (2.3, 10.1) | 39 | 7.1 (5.2, 9.7) | 5.4 (2.3, 10.1) | Reference |
| **ESKD before age 60^2^, N=1169** |  |  |  |  |  |  |
| Rapid decliner (n=102) | 551 | 4.3 (3.0, 7.4) | 48 | 87.2 (70.4, 107.9) | 5.2 (3.8, 8.6) | 7.2 (5.1, 10.2) |
| Non-rapid decliner (n=1067) | 5774 | 4.3 (1.9, 8.0) | 77 | 13.3 (10.7, 16.6) | 5.8 (3.1, 8.6) | Reference |
| **ESKD before age 62^2^, N=1220** |  |  |  |  |  |  |
| Rapid decliner (n=104) | 583 | 4.3 (3.0, 7.4) | 48 | 82.3 (65.9, 102.8) | 4.7 (3.0, 7.6) | 7.2 (5.2, 10.2) |
| Non-rapid decliner (n=1116) | 7091 | 5.3 (2.3, 9.7) | 88 | 12.4 (10.1, 15.2) | 5.3 (2.3, 9.7) | Reference |
| **ESKD before age 63^2^, N=1254** |  |  |  |  |  |  |
| Rapid decliner (n=107) | 594 | 4.7 (3.0, 7.6) | 48 | 80.8 (64.7, 100.9) | 4.6 (3.0, 7.5) | 7.2 (5.1, 10.1) |
| Non-rapid decliner (n=1147) | 7287 | 5.3 (2.3, 9.7) | 89 | 12.2 (10.0, 15.0) | 5.3 (2.3, 9.7) | Reference |
| **Mortality^3^, N=1744** |  |  |  |  |  |  |
| Rapid decliner (n=125) | 926 | 6.0 (3.4, 11.5) | 19 | 20.5 (13.2, 31.9) | 6.7 (2.5, 8.3) | 0.9 (0.6, 1.5) |
| Non-rapid decliner (n=1619) | 10,661 | 5.6 (2.5, 9.9) | 232 | 21.8 (19.2, 24.7) | 4.4 (1.7, 8.4) | Reference |
| ^1^Patients were followed from baseline to ESKD, mortality, disenrollment, or end of study (1/1/2020), whichever came first. ^2^Restricted to patients who were younger than age 53, 60, 62, or 63 at baseline. ^3^Patients were followed from baseline to mortality, disenrollment, or end of study (1/1/2020), whichever came first. *95% CI based on robust Poisson regression | | | | | | |

**Supplemental Table 2. Total Patient Follow-up Time, Events and Incidence Rate of ESKD per 1,000 Person-Years**

**Supplemental Table 3. ESKD within 5 Years of ADPKD Diagnosis by Non-Rapid Decliner vs. Rapid Decliner Group**

|  | Non-Rapid Decliner  (N = 895) | Rapid Decliner  (N = 81) | *p-*value |
| --- | --- | --- | --- |
| ESKD within 5-years, n (%) |  |  | <0.01 |
| No | 832 (93.0) | 50 (61.7) |  |
| Yes | 63 (7.0) | 31 (38.3) |  |

**Supplementary Figures**

**Supplementary Figure 1.** **Consort diagram**


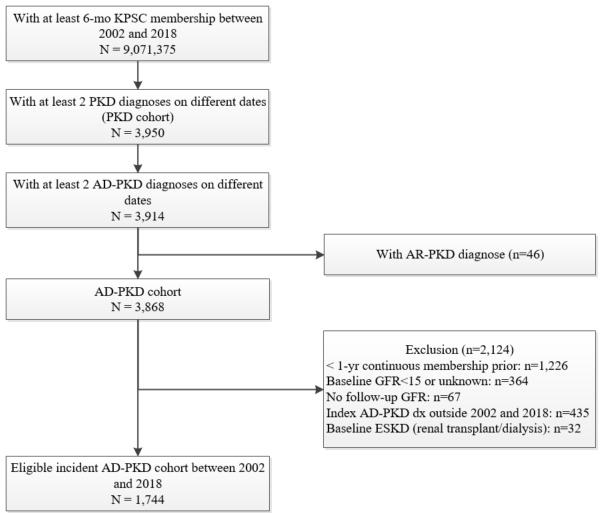


**Supplementary Figure 2.** **Cumulative incidence (CI) plots for ESKD, ESKD < 60 years, and mortality**


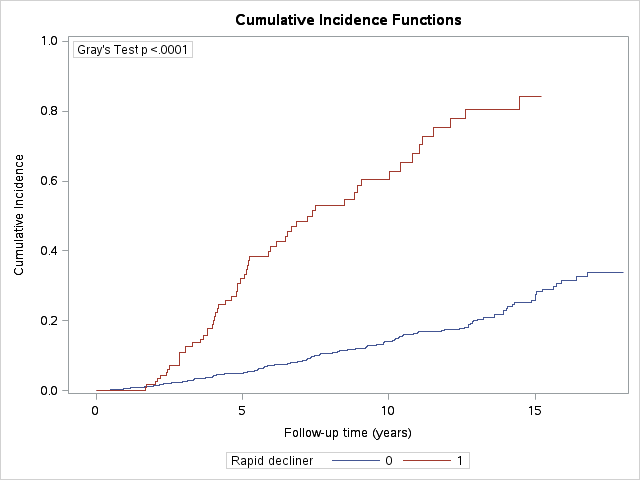

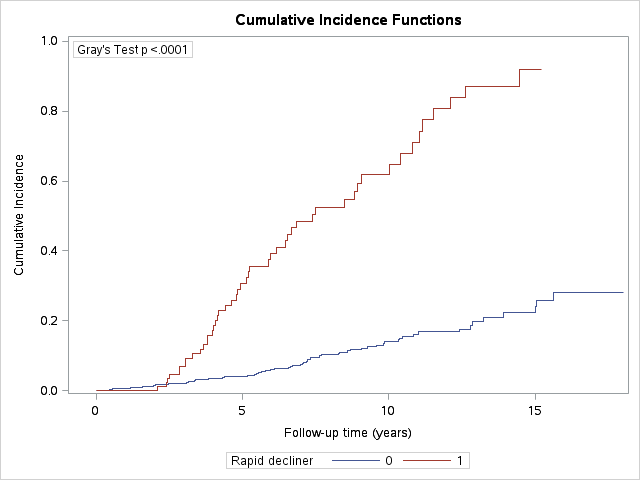


CI for ESKD (mortality as competing risk) CI for ESKD < age 60 (mortality as competing risk)


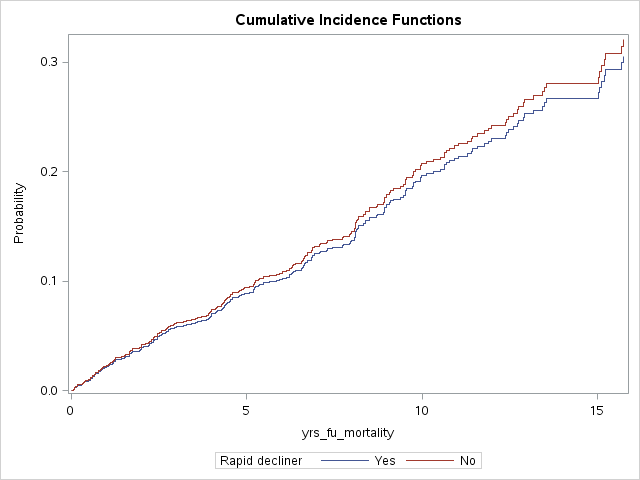


CI for mortality
